# Supplementary material for: Anticancer Effects of Thymoquinone through the Antioxidant Activity, Upregulation of Nrf2, and Downregulation of PD-L1 in Triple-Negative Breast Cancer Cells
Source: Nutrients. 2022 Nov 13;14(22):4787. doi: 10.3390/nu14224787 (PMC9695946; doi:10.3390/nu14224787)
Supplement: Supplementary file 1 [file nutrients-14-04787-s001.zip › nutrients-1950213-supplementary.pdf]

1. Nrf2 protein expression in TQ treated INF-Y stimulated MDA-MB-231 TNBC cells

A.

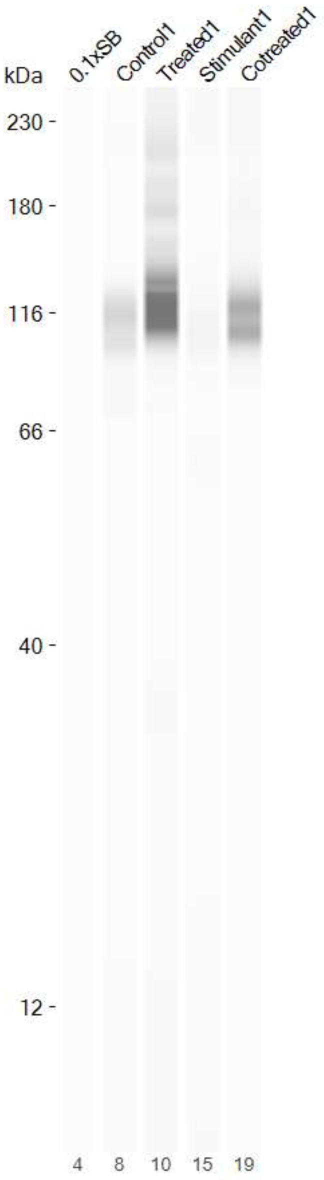

Peaks | Capillaries

Sample	Prim...	Cap	Peak	Name	Positi...	MW (...	Height	Area	% Area	Width	S/N	Baseline
Control1	Nrf2	8	1		627	116	308.1	5587.7		17.0	40.0	40.8
Treated1	Nrf2	10	1		624	121	477.6	2030...		39.9	11...	55.9
Stimulant1	Nrf2	15	1		620	104	144.9	1604.5		10.4	27.7	56.6
Cotreated1	Nrf2	19	1		624	109	351.7	1187...		31.7	79.4	33.8

B.

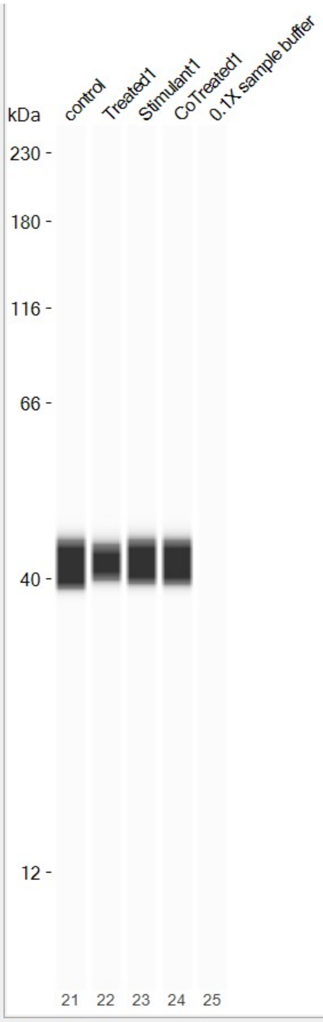

Peaks | Capillaries

Sample	Prim...	Cap	Peak	Name	Positi...	MW (...	Height	Area	% Area	Width	S/N	Baseline
control	GAP...	21	1		543	42	2010...	1684...		7.9	13...	23.3
Treated1	GAP...	22	1		543	42	7650.5	6383...		7.8	35...	20.5
Stimula...	GAP...	23	1		543	42	1466...	1204...		7.7	74...	35.0
CoTreat...	GAP...	24	1		545	42	1493...	1245...		7.8	15...	7.5
0.1X sa...	GAP...	25	1		544	41	5.8	265.9		43.0	39.5	2.2

C.

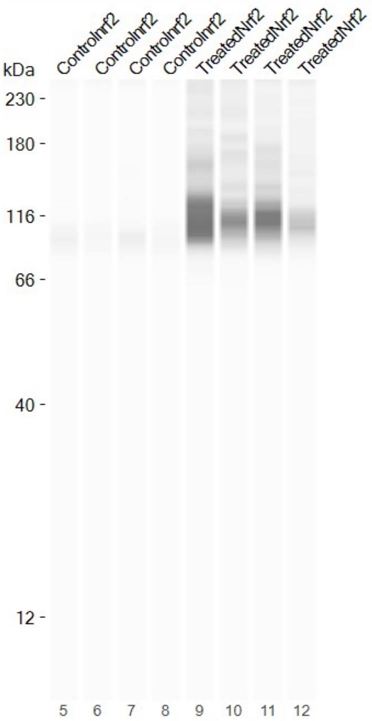

Peaks | Capillaries

Sample	Prim...	Cap	Peak	Name	Positi...	MW (...	Height	Area	% Area	Width	S/N	Baseline
Controlnrf2	NrF2	5	1		613	98	456.1	6761.5		13.9	93.6	22.3
Controlnrf2	NrF2	6	1		613	99	218.3	9214.2		39.7	50.6	33.2
Controlnrf2	NrF2	7	1		610	99	369.5	8555.7		21.8	77.7	52.6
Controlnrf2	NrF2	8	1		611	97	212.2	11205.3		49.6	54.4	43.3
TreatedNrf2	NrF2	9	1		526	38	36.3	1088.9		28.2	14.3	65.1
TreatedNrf2	NrF2	9	2		618	108	1623.9	38383.7		22.2	23...	59.6
TreatedNrf2	NrF2	9	3		650	161	841.9	28838.9		32.2	58.8	59.8
TreatedNrf2	NrF2	10	1		620	114	1134.6	36748.4		30.4	19...	62.2
TreatedNrf2	NrF2	10	2		674	213	553.4	22135.8		37.6	35.7	61.4
TreatedNrf2	NrF2	11	1		574	57	36.5	944.9		24.3	15.0	71.1
TreatedNrf2	NrF2	11	2		624	111	1389.4	33070.8		22.4	18...	68.3
TreatedNrf2	NrF2	11	3		652	163	677.9	20287.1		28.1	49.8	67.0
TreatedNrf2	NrF2	12	1		580	60	25.3	1159.3		43.0	14.2	59.1
TreatedNrf2	NrF2	12	2		623	107	619.9	14756.0		22.4	16...	54.6
TreatedNrf2	NrF2	12	3		651	157	409.6	23203.2		53.2	29.3	52.5

Supplement Figure S1. Nrf2 protein expression in TQ treated INF-Y stimulated MDA-MB-231 TNBC cells. Following the overnight incubation period, cells were equally treated with 15 µM of TQ for 24. A. Blot for Nrf2 protein expression in control, treated, stimulated and cotreated MDA-MB-231 cell. B. Blot for protein expression of GAPDH used for normalization. C. Blot for Nrf2 protein expression in control and TQ treated MDA-MB-231 cell in triplicates.

2. Nrf2 protein expression in TQ treated INF-Y stimulated MDA-MB-468 TNBC cell

A.

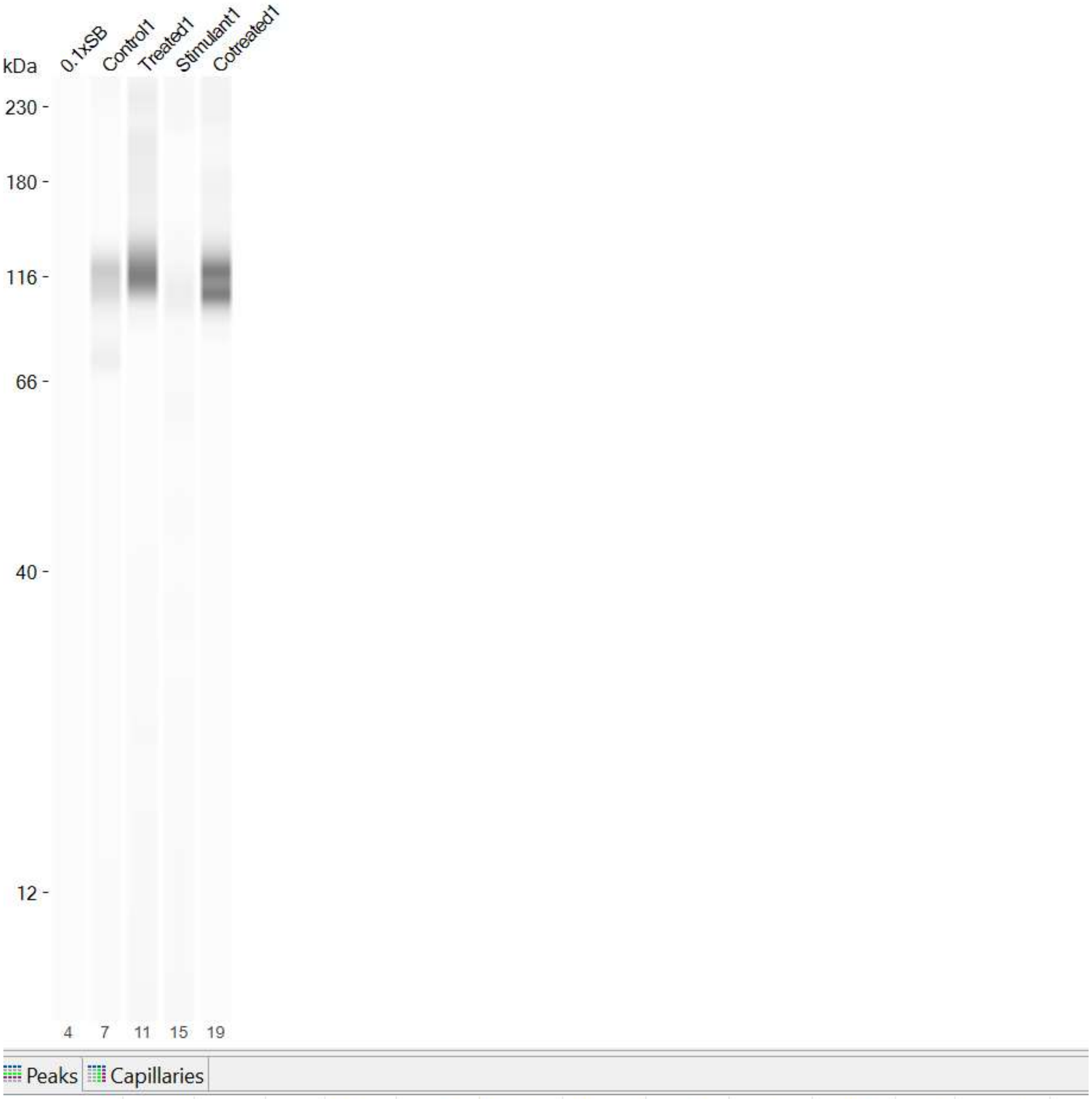

Sample	Prim...	Cap	Peak	Name	Positi...	MW (...	Height	Area	% Area	Width	S/N	Baseline
Control1	Nrf2	7	1		627	119	242.3	5333.3		20.7	42.9	39.3
Treated1	Nrf2	11	1		626	114	523.4	4292.3		7.7	79.7	43.4
Stimula...	Nrf2	15	1		620	104	144.9	1604.5		10.4	27.7	56.6
Cotreat...	Nrf2	19	1		624	109	351.7	1187...		31.7	79.4	33.8

B.

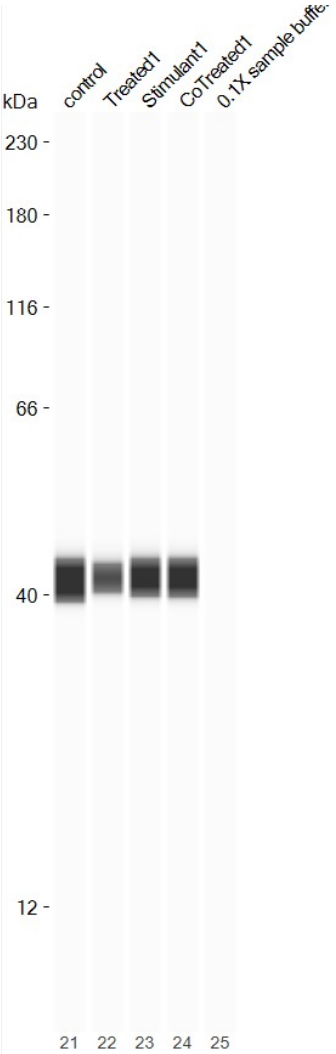

Peaks | Capillaries

Sample	Prim...	Cap	Peak	Name	Positi...	MW (...	Height	Area	% Area	Width	S/N	Baseline
control	GAP...	21	1		543	42	2010...	1684...		7.9	13...	23.3
Treated1	GAP...	22	1		543	42	7650.5	6383...		7.8	35...	20.5
Stimulant1	GAP...	23	1		543	42	1466...	1204...		7.7	74...	35.0
CoTreated1	GAP...	24	1		545	42	1493...	1245...		7.8	15...	7.5
0.1X samp...	GAP...	25	1		544	41	5.8	265.9		43.0	39.5	2.2

C.

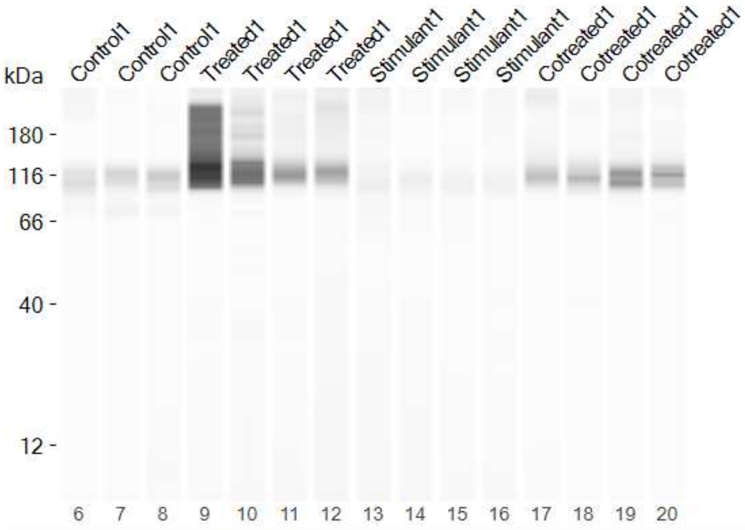

Peaks | Capillaries

Sample	Prim...	Cap	Peak	Name	Positi...	MW (...	Height	Area	% Area	Width	S/N	Baseline
Control1	Nrf2	7	1		627	119	242.3	5333.3		20.7	42.9	39.3
Control1	Nrf2	8	1		627	116	308.1	5587.7		17.0	40.0	40.8
Treated1	Nrf2	9	1		628	130	1364.4	36336.3		25.0	19...	48.3
Treated1	Nrf2	9	2		669	204	639.4	16833.7		24.7	89.6	43.5
Treated1	Nrf2	10	1		624	121	477.6	20300.1		39.9	11...	55.9
Treated1	Nrf2	11	1		626	114	523.4	4292.3		7.7	79.7	43.4
Treated1	Nrf2	12	1		627	121	264.7	9873.7		35.0	58.5	75.7
Treated1	Nrf2	12	2		673	224	117.2	4308.0		34.5	18.4	66.8
Stimula...	Nrf2	13	1		619	103	103.8	4638.0		42.0	23.5	62.7
Stimula...	Nrf2	14	1		624	113	148.9	1786.9		11.3	25.7	64.7
Stimula...	Nrf2	15	1		620	104	144.9	1604.5		10.4	27.7	56.6
Stimula...	Nrf2	16	1		617	108	84.6	3481.0		38.7	24.3	55.6
Cotreat...	Nrf2	17	1		626	113	317.1	6886.4		20.4	51.6	36.0
Cotreat...	Nrf2	18	1		625	114	302.1	9042.7		28.1	62.8	29.2
Cotreat...	Nrf2	18	2		672	216	87.9	2555.2		27.3	19.5	23.8
Cotreat...	Nrf2	19	1		624	109	351.7	11878.9		31.7	79.4	33.8
Cotreat...	Nrf2	20	1		623	107	320.0	8677.4		25.5	44.6	30.5

Figure S2. Nrf2 protein expression in TQ treated INF-Y stimulated MDA-MB-468TNBC cells. Following the overnight incubation period, cells were equally treated with 15 µM of TQ for 24. A. Blot for Nrf2 protein expression in control, treated, stimulated and cotreated MDA-MB-468 cell. B. Blot for protein expression of GAPDH used for normalization. C. . Blot for Nrf2 protein expression in control, treated, stimulated and cotreated MDA-MB-468 cell in different replicates.

3. PD-L1 protein expression in TQ treated INF-Y stimulated MDA-MB-231 TNBC cells

A

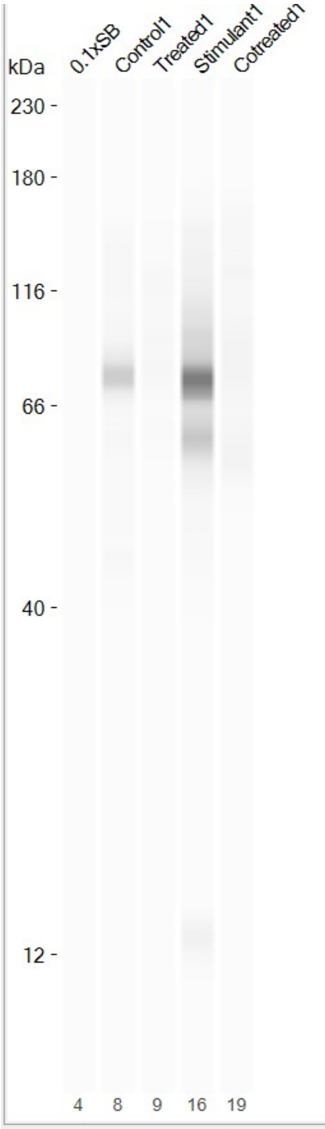

Peaks Capillaries

Sample	Prim...	Cap	Peak	Name	Positi...	MW (...	Height	Area	% Area	Width	S/N	Baseline
Control1	PDL-1	8	1		610	78	1726.5	2523...		13.7	13...	126.4
Stimulant1	PDL-1	16	1		457	14	500.9	1595...		29.9	91.6	98.2
Stimulant1	PDL-1	16	2		591	62	679.1	2207...		30.5	10...	137.5
Stimulant1	PDL-1	16	3		607	78	2092.5	7529...		33.8	22...	144.9
Cotreated1	PDL-1	19	1		589	59	556.4	1368...		23.1	45.0	121.4
Cotreated1	PDL-1	19	2		617	82	726.7	2135...		27.6	27.2	124.8
Cotreated1	PDL-1	19	3		655	146	541.5	1569...		27.2	29.8	128.0

B.

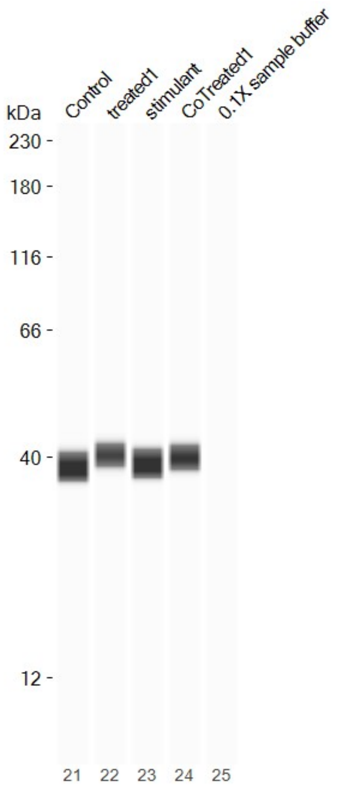

Peaks | Capillaries

Sample	Prim...	Cap	Peak	Name	Positi...	MW (...	Height	Area	% Area	Width	S/N	Baseline
Control	GAP...	21	1		500	26	208.8	2000.0		9.0	68.2	46.3
Control	GAP...	21	2		543	39	2265...	1971...		8.2	91...	46.7
Control	GAP...	21	3		632	114	2711.8	3994...		13.8	24...	47.7
Control	GAP...	21	4		653	155	2279.6	3132...		12.9	19...	47.9
treated1	GAP...	22	1		550	40	1394...	1194...		8.0	43...	46.0
treated1	GAP...	22	2		613	82	545.2	7492.3		12.9	26.6	49.3
treated1	GAP...	22	3		625	98	805.1	1229...		14.3	29.4	50.0
treated1	GAP...	22	4		653	147	1214.2	2036...		15.8	10...	51.7
stimulant	GAP...	23	1		500	26	695.8	7015.5		9.5	23...	17.0
stimulant	GAP...	23	2		543	39	2276...	1957...		8.1	83...	14.0
stimulant	GAP...	23	3		606	77	1462.0	2044...		13.1	35.5	12.4
stimulant	GAP...	23	4		622	109	1990.1	2903...		13.7	30.4	12.4
stimulant	GAP...	23	5		647	145	1822.7	2554...		13.2	13...	12.8
CoTreated1	GAP...	24	1		548	40	1639...	1396...		8.0	59...	30.8
CoTreated1	GAP...	24	2		621	100	1001.6	1360...		12.8	43.1	34.1
CoTreated1	GAP...	24	3		648	141	1045.4	1392...		12.5	10...	35.3
0.1X sampl...	GAP...	25	1		547	39	46.9	1557.9		31.2	12...	1.6

C.

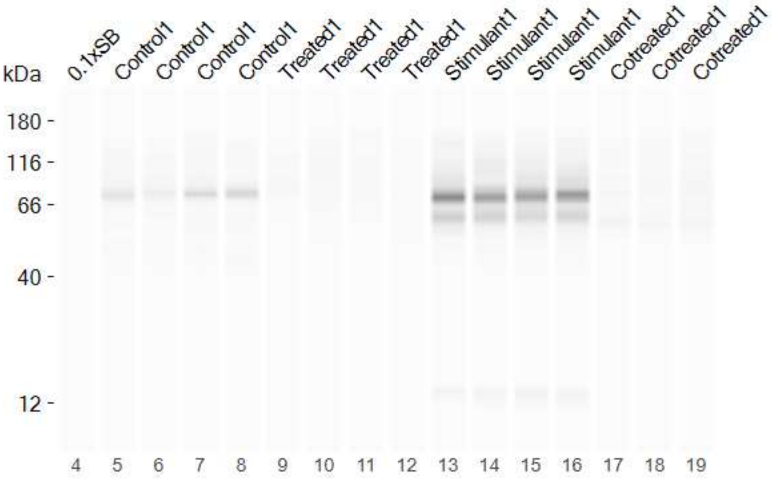

Peaks | Capillaries

Sample	Primary	Cap	Peak	Name	Positi...	MW (...	Height	Area	% Area	Width	S/N	Baseline
Control1	PDL-1	5	1		607	77	1052.9	39912.7		35.6	10...	137.3
Control1	PDL-1	6	1		607	80	1021.7	24907.4		22.9	67.5	141.1
Control1	PDL-1	7	1		608	78	1515.7	26612.8		16.5	15...	153.2
Control1	PDL-1	8	1		610	78	1726.5	25239.6		13.7	13...	126.4
Stimula...	PDL-1	13	1		459	14	735.6	10191.1		13.0	74.6	167.4
Stimula...	PDL-1	13	2		495	25	48.0	699.0		13.7	11.3	200.8
Stimula...	PDL-1	13	3		515	31	54.5	793.3		13.7	12.9	219.5
Stimula...	PDL-1	13	4		589	61	1597.3	23869.6		14.0	89.4	263.6
Stimula...	PDL-1	13	5		605	75	2794.7	46755.8		15.7	21...	267.0
Stimula...	PDL-1	13	6		647	143	841.0	13838.9		15.5	32.7	269.0
Stimula...	PDL-1	14	1		458	14	472.8	13721.9		27.3	60.5	140.1
Stimula...	PDL-1	14	2		590	61	758.0	21641.3		26.8	73.4	188.0
Stimula...	PDL-1	14	3		607	75	2108.2	64190.9		28.6	14...	187.1
Stimula...	PDL-1	15	1		457	14	541.3	15054.0		26.1	91.9	142.6
Stimula...	PDL-1	15	2		590	62	494.5	15831.1		30.1	73.1	227.9
Stimula...	PDL-1	15	3		607	76	2037.5	68402.3		31.5	18...	235.5
Stimula...	PDL-1	16	1		457	14	500.9	15952.4		29.9	91.6	98.2
Stimula...	PDL-1	16	2		591	62	679.1	22075.2		30.5	10...	137.5
Stimula...	PDL-1	16	3		607	78	2092.5	75298.2		33.8	22...	144.9
Cotreat...	PDL-1	17	1		591	60	700.6	21823.3		29.3	47.0	131.4
Cotreat...	PDL-1	18	1		652	143	599.2	12789.7		20.1	33.5	97.5
Cotreat...	PDL-1	19	1		589	59	556.4	13689.6		23.1	45.0	121.4
Cotreat...	PDL-1	19	2		617	82	726.7	21359.7		27.6	27.2	124.8
Cotreat...	PDL-1	19	3		655	146	541.5	15697.8		27.2	29.8	128.0

Figure 3. PD-L1 protein expression in TQ treated INF-Y stimulated MDA-MB-231 TNBC cells. Following the overnight incubation period, cells were equally treated with 15 µM of TQ for 24. A. Blot for PD-L1 protein expression in control, treated, stimulated and cotreated MDA-MB-231 cell. B. Blot for protein expression of GAPDH used for normalization.

4. PD-L1 protein expression in TQ treated INF-Y stimulated MDA-MB-468 TNBC cells

A.

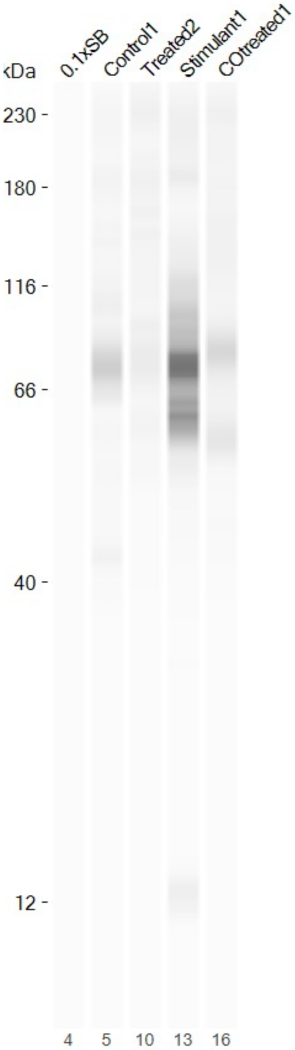

Peaks | Capillaries

Sample	Prim...	Cap	Peak	Name	Positi...	MW (...	Height	Area	% Area	Width	S/N	Baseline
Control1	PDL...	5	1		544	44	377.6	1540...		38.3	40.6	131.5
Control1	PDL...	5	2		603	79	987.5	3479...		33.1	70.8	183.2
Control1	PDL...	5	3		658	186	381.8	1728...		42.5	22.1	237.0
Treated2	PDL...	10	1		604	78	689.8	1764...		24.0	24.9	249.5
Treated2	PDL...	10	2		682	231	421.0	1026...		22.9	23.6	295.6
Stimula...	PDL...	13	1		443	13	484.5	1026...		19.9	39.4	155.2
Stimula...	PDL...	13	2		587	62	935.5	2473...		24.8	50.3	214.5
Stimula...	PDL...	13	3		603	78	1948.6	5575...		26.9	88.0	218.7
COtreat...	PDL...	16	1		581	60	444.4	1523...		32.2	49.0	205.5
COtreat...	PDL...	16	2		608	82	750.1	2586...		32.4	56.3	229.2
COtreat...	PDL...	16	3		683	231	0.0	0.3		31.9	24.5	296.9

B.

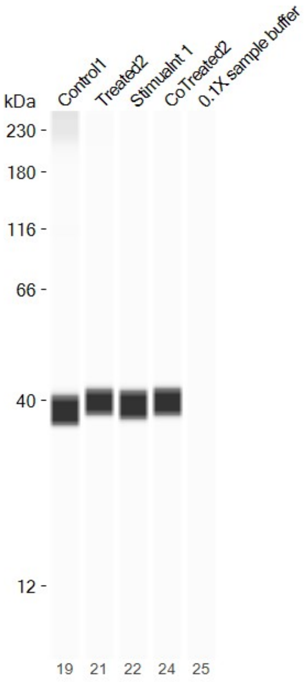

Peaks Capillaries

Sample	Prim...	Cap	Peak	Name	Positi...	MW (...	Height	Area	% Area	Width	S/N	Baseline
Control1	GAP...	19	1		491	26	319.9	3073.5		9.0	69.7	96.8
Control1	GAP...	19	2		532	38	1933...	1758...		8.5	52...	101.2
Control1	GAP...	19	3		627	115	3057.8	3373...		10.4	26...	107.8
Control1	GAP...	19	4		678	226	1011...	1191...		11.1	80...	109.9
Control1	GAP...	19	5		690	249	1165...	1427...		11.5	10...	110.3
Treated2	GAP...	21	1		539	40	1534...	1306...		8.0	37...	89.3
Treated2	GAP...	21	2		608	90	1086.7	1984...		17.2	24.6	101.5
Treated2	GAP...	21	3		648	158	1444.8	2823...		18.4	31.1	106.9
Treated2	GAP...	21	4		682	229	3295.1	7269...		20.7	24...	110.9
Stimual...	GAP...	22	1		490	26	441.2	4471.8		9.5	16...	64.7
Stimual...	GAP...	22	2		536	39	1795...	1632...		8.5	72...	90.9
Stimual...	GAP...	22	3		582	60	366.1	4256.2		10.9	20.3	124.1
Stimual...	GAP...	22	4		628	117	1249.5	1711...		12.9	67.4	162.2
Stimual...	GAP...	22	5		646	149	1098.0	1456...		12.5	44.8	178.0
Stimual...	GAP...	22	6		678	225	1891.7	2915...		14.5	26...	206.8
CoTreat...	GAP...	24	1		541	40	1628...	1436...		8.3	73...	83.9
CoTreat...	GAP...	24	2		620	98	619.3	8284.3		12.6	57.7	156.1
CoTreat...	GAP...	24	3		683	225	1635.9	2634...		15.1	23...	227.4
0.1X sa...	GAP...	25	1		540	39	13.2	1279.6		91.3	26...	0.6

C.

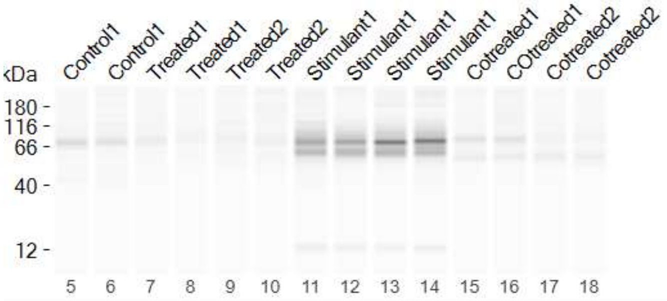

Peaks | Capillaries

Sample	Primary	Cap	Peak	Name	Positi...	MW (...	Height	Area	% Area	Width	S/N	Baseline
Control1	PDL-1	5	1		544	44	377.6	15401.7		38.3	40.6	131.5
Control1	PDL-1	5	2		603	79	987.5	34799.8		33.1	70.8	183.2
Control1	PDL-1	5	3		658	186	381.8	17284.3		42.5	22.1	237.0
Control1	PDL-1	6	1		602	80	597.3	62888.2		98.9	77.5	195.8
Treated1	PDL-1	7	1		607	84	818.3	24417.0		28.0	33.1	169.1
Treated2	PDL-1	10	1		604	78	689.8	17641.9		24.0	24.9	249.5
Treated2	PDL-1	10	2		682	231	421.0	10267.0		22.9	23.6	295.6
Stimula...	PDL-1	11	1		441	13	359.4	14058.2		36.8	53.8	168.5
Stimula...	PDL-1	11	2		585	62	331.5	14928.7		42.3	58.5	267.7
Stimula...	PDL-1	11	3		604	78	1612.3	76080.5		44.3	50.2	275.0
Stimula...	PDL-1	12	1		441	12	686.2	10324.7		14.1	52.3	140.8
Stimula...	PDL-1	12	2		585	63	1715.2	27172.5		14.9	59.9	244.0
Stimula...	PDL-1	12	3		603	79	1909.1	30474.4		15.0	59.9	255.6
Stimula...	PDL-1	12	4		683	239	426.3	6570.5		14.5	26.6	296.2
Stimula...	PDL-1	13	1		443	13	484.5	10267.7		19.9	39.4	155.2
Stimula...	PDL-1	13	2		587	62	935.5	24733.1		24.8	50.3	214.5
Stimula...	PDL-1	13	3		603	78	1948.6	55753.5		26.9	88.0	218.7
Stimula...	PDL-1	14	1		444	13	441.3	13867.3		29.5	54.9	158.5
Stimula...	PDL-1	14	2		589	62	639.7	24419.3		35.9	40.3	265.7
Stimula...	PDL-1	14	3		607	81	1787.3	67359.7		35.4	77.3	280.6
Stimula...	PDL-1	14	4		662	187	685.5	25012.9		34.3	21.4	327.2
Stimula...	PDL-1	14	5		679	227	0.0	0.3		29.9	28.2	341.8
Cotreat...	PDL-1	15	1		442	12	79.2	1985.3		23.6	12.3	107.2
Cotreat...	PDL-1	15	2		580	58	666.1	16145.8		22.8	53.5	169.4
Cotreat...	PDL-1	15	3		609	87	928.8	26715.9		27.0	77.3	185.5
Cotreat...	PDL-1	15	4		681	222	430.1	12300.1		26.9	31.0	227.9
COtreat...	PDL-1	16	1		581	60	444.4	15235.7		32.2	49.0	205.5
COtreat...	PDL-1	16	2		608	82	750.1	25864.7		32.4	56.3	229.2
COtreat...	PDL-1	16	3		683	231	0.0	0.3		31.9	24.5	296.9
Cotreat...	PDL-1	17	1		584	59	894.6	15593.6		16.4	41.4	134.2
Cotreat...	PDL-1	17	2		608	81	718.2	12669.9		16.6	19.1	136.6
Cotreat...	PDL-1	17	3		680	231	447.2	7774.3		16.3	20.5	141.9
Cotreat...	PDL-1	18	1		583	59	958.0	14968.0		14.7	54.6	107.8

D.

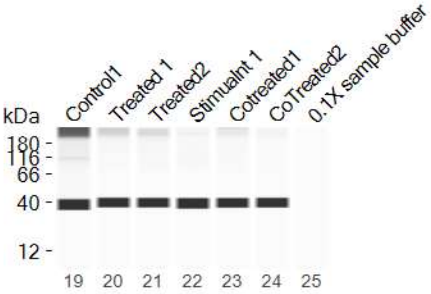

Peaks | Capillaries

Sample	Primary	Cap	Peak	Name	Positi...	MW (...	Height	Area	% Area	Width	S/N	Baseline
Control1	GAPDH	19	1		491	26	319.9	3073.5		9.0	69.7	96.8
Control1	GAPDH	19	2		532	38	1933...	1758672.6		8.5	52...	101.2
Control1	GAPDH	19	3		627	115	3057.8	33739.1		10.4	26...	107.8
Control1	GAPDH	19	4		678	226	1011...	119181.5		11.1	80...	109.9
Control1	GAPDH	19	5		690	249	1165...	142728.4		11.5	10...	110.3
Treated 1	GAPDH	20	1		538	40	1562...	1340560.5		8.1	31...	107.5
Treated 1	GAPDH	20	2		648	163	1383.8	23262.3		15.8	27.5	126.4
Treated 1	GAPDH	20	3		689	236	3906.7	80083.9		19.3	20...	131.0
Treated2	GAPDH	21	1		539	40	1534...	1306211.3		8.0	37...	89.3
Treated2	GAPDH	21	2		608	90	1086.7	19844.4		17.2	24.6	101.5
Treated2	GAPDH	21	3		648	158	1444.8	28237.5		18.4	31.1	106.9
Treated2	GAPDH	21	4		682	229	3295.1	72694.2		20.7	24...	110.9
Stimual...	GAPDH	22	1		490	26	441.2	4471.8		9.5	16...	64.7
Stimual...	GAPDH	22	2		536	39	1795...	1632468.4		8.5	72...	90.9
Stimual...	GAPDH	22	3		582	60	366.1	4256.2		10.9	20.3	124.1
Stimual...	GAPDH	22	4		628	117	1249.5	17118.1		12.9	67.4	162.2
Stimual...	GAPDH	22	5		646	149	1098.0	14568.9		12.5	44.8	178.0
Stimual...	GAPDH	22	6		678	225	1891.7	29158.2		14.5	26...	206.8
Cotreat...	GAPDH	23	1		540	39	1673...	1430883.5		8.0	59...	64.7
Cotreat...	GAPDH	23	2		619	99	1024.9	14406.8		13.2	30.5	75.0
Cotreat...	GAPDH	23	3		649	155	1258.2	18625.8		13.9	40.3	78.1
Cotreat...	GAPDH	23	4		679	221	2183.5	31538.0		13.6	19...	80.8
CoTreat...	GAPDH	24	1		541	40	1628...	1436378.5		8.3	73...	83.9
CoTreat...	GAPDH	24	2		620	98	619.3	8284.3		12.6	57.7	156.1
CoTreat...	GAPDH	24	3		683	225	1635.9	26348.9		15.1	23...	227.4
0.1X sa...	GAPDH	25	1		540	39	13.2	1279.6		91.3	26...	0.6

Figure S4. PD-L1 protein expression in TQ treated INF-Y stimulated MDA-MB-468 TNBC cells. Following the overnight incubation period, cells were equally treated with 15 µM of TQ for 24. A. Blot for PD-L1 protein expression in control, treated, stimulated and cotreated MDA-MB-468 cell. B. Blot for protein expression of GAPDH used for normalization. C. Blot for PD-L1 protein expression in control, treated, stimulated and cotreated MDA-MB-468 cell. D. Blot for protein expression of GAPDH used for normalization.
